# A systematic review of the literature on the impact of the Seguro Popular

**DOI:** 10.1186/s12961-022-00839-w

**Published:** 2022-04-18

**Authors:** M. A. Colchero, R. Gómez, S. Bautista-Arredondo

**Affiliations:** Center for Health Systems Research, Instituto Nacional de Salud Pública, Universidad No. 655 Colonia Santa María Ahuacatitlán, Cerrada Los Pinos y Caminera, C.P. 62100 Cuernavaca, Morelos Mexico

**Keywords:** Seguro Popular, Universal health coverage, Impact evaluation, Mexico

## Abstract

**Background:**

The Seguro Popular (SP) was launched in 2004 to increase access to healthcare and reduce catastrophic expenditures among the Mexican population. To document the evidence on its effectiveness, we conducted a systematic review of impact evaluations of the SP.

**Methods:**

We included papers using rigorous quasi-experimental designs to assess the effectiveness of the SP. We evaluated the quality of each study and presented the statistical significance of the effects by outcome category.

**Results:**

We identified 26 papers that met the inclusion criteria. Sixteen studies that evaluated the impact of SP on financial protection found consistent and statistically significant positive effects in 55% of the 65 outcomes analyzed. Nine studies evaluating utilization of health services for the general and infant populations found effectiveness on 40% of 30 outcomes analyzed. Concerning screening services for hypertension, diabetes, and cervical and prostate cancer, we found three studies evaluating 14 outcomes and finding significant effects on 50% of them. Studies looking at the impact of SP on diabetes, hypertension, and general health care and treatment evaluated 19 outcomes and found effects on 21% of them. One study assessed five diabetes monitoring services and found positive effects on four of them. The only study on morbidity and mortality found positive results on three of the four outcomes of interest.

**Conclusion:**

We found mixed evidence on the impact of SP on financial protection, healthcare utilization, morbidity and mortality. In the 26 studies included in this review, researchers found positive effects in roughly half of the outcomes and null results on the rest.

**Supplementary Information:**

The online version contains supplementary material available at 10.1186/s12961-022-00839-w.

## Background

As in many Latin American countries, segmentation, inefficiencies and inequalities hinder the Mexican health system's potential [1]. Employees in the formal sector, either private or government, have access to social security and health insurance. The rest of the population has access to partially funded services offered by Ministry of Health facilities, with no access to other public health care services. Most of the population has access to private health care though primary care providers and medical offices in pharmacies, with more limited access to private hospitals. Overlaps in coverage given labour rotation create multiple duplications and require massive administration to function [1]. Additionally, given the enormous mobility of workers between formal and informal jobs, the bureaucracy involved in updating their status is a barrier to healthcare [2]. The configuration of the health system creates inequalities as well. By 2003, private health expenditures represented 58% of total health expenditures in the country [3].

To address some of these flaws, in 2004, the Mexican government introduced the System of Social Protection in Health to provide health insurance to the population without social security and reduce out-of-pocket expenditures [4]. Among other elements, the reform established a funding mechanism called Seguro Popular (SP), or Popular Health Insurance, designed to increase free access to a predetermined set of interventions and services to reduce catastrophic expenditures for the uninsured.

By 2012, the SP had enrolled 52 million people, or about 75% of the uninsured population [5]. This achievement was followed by increased use of healthcare services and reduced out-of-pocket expenditures [6]. In the second half of the 2000s and most of the 2010s, multiple studies evaluated the impact of the SP on several dimensions of health, healthcare utilization, and financial protection, as we describe in this study. SP became one of the better-documented examples of access to universal health coverage (UHC) in a middle-income country; nonetheless, as we show in this paper, many of these studies lacked robust methods to establish causality and identify SP's attributable effects. Following the health reform introduced by the 2018–2024 federal administration, the SP was abolished in 2019.

Evaluating the impact of the SP is not a simple task. The SP started in a pilot phase in five states (Aguascalientes, Campeche, Colima, Jalisco and Tabasco) in October 2001 but was formally implemented in 2004 when it legaly constituted the operating arm of the System for Social Protection in Health [7]. Furthermore, affiliation to SP was voluntary, ruling out the possibility of comparing affiliates with non-affiliates for the evaluation due to selection bias as people choosing to affiliate may be different compared to non-affiliates in observed and unobserved characteristics that could be associated with outcomes of interest. Studies addressing selection bias and other potential confounding factors used quasi-experimental approaches by exploiting the program's variability in time of initiation and level of penetration across states. Results from studies failing to address this potential bias result in biased estimations. Studies addressing the selection bias and other potential confounding factors used a quasi-experimental design with no experimental groups by exploiting the programme's variability in time and penetration by state.

The SP was successful in enrolling the uninsured population [8]. However, the picture arising from the body of evidence is less clear concerning the impact on the beneficiaries' access to healthcare and health status. Despite being one of the better-documented UHC programmes globally, there are no published systematic reviews on the SP's impact, which is an important task given some of the studies' methodological limitations. Our study's objective was to conduct a systematic review of published studies that assessed the impact of the SP on healthcare utilization, screening, access to treatment, financial protection and health outcomes. Our review focuses on papers that used econometric methods to explicitly address the potential selection biases, excluding those that failed to use methods to address this bias.

## Methods

### Information sources and search strategy

For this systematic review, we conducted a search strategy in MEDLINE (PubMed) and the Latin American and Caribbean Literature in Health Sciences (LILACS). The strategy combined keywords, Boolean operators and proximity operators initially designed for MEDLINE and adapted to LILACS. The search was conducted in July and August 2020 and updated in February 2022 to identify the peer-reviewed evaluations of the SP. In the second phase of the search, we reviewed the reference lists of the studies selected through the first strategy to identify additional references. We used the following keywords: seguro popular, health insurance, health reform, Mexican health insurance**,** and the MeSH term “Health Care Reform”**.** The final search algorithms were as follows: “((Seguro popular) OR (popular health insurance) OR (popular insurance) OR (public insurance) OR (health reform)) AND ((evaluation) OR (effect) OR (assessment) OR (impact)) AND (Mexico ((United States) OR (European)) Filters: from 2002 – 2022” for MEDLINE. The LILACS algorithm was as follows: “(Seguro popular OR popular insurance OR popular health insurance AND evaluation OR impact OR effect AND Mexico (yearcluster:[2002 TO 2020])”.

### Selection process (inclusion and exclusion criteria)

We searched for original, quantitative studies published between 2002 and 2022. We excluded studies that were not peer-reviewed, such as the grey literature, comments, theses and protocols, and papers not written in English or Spanish. Given that SP's affiliation was voluntary, there was a potential selection bias in the analyses that simply compared enrolled and unenrolled households. Those enrolled may be systematically different from unenrolled households in characteristics associated with the outcomes of interest. For example, they may be sicker, or they may be more cautious and healthier. It is impossible to guess, and therefore measure, the magnitude and direction of this potential bias. Thus, evaluations that fail to address it may report biased results. We excluded papers that did not use methods to explicitly address this potential bias [4], such as instrumental variables, propensity score matching or regression discontinuity. We also included studies that used interrupted time series analyses with and without a comparison group.

### Quality assessment of studies

We applied the Specialist Unit for Review Evidence (SURE) to evaluate the quality of each paper. SURE evaluates 11 key features of a paper: study design; study question; settings, locations, and dates; participant selection; characteristics of participants; appropriate outcome and exposure measures; sample size; adequate description of the methods and results; sponsorship/conflict of interest and limitations. For each item, we added specific aspects to evaluate the quality of the methods used for impact evaluation (Additional file 1). Two researchers evaluated each paper separately, and differences were discussed between authors until consensus was reached. Each item in SURE scores 1, so the maximum score is 11.

### Data extraction

Using the Mendeley Desktop version 1.19.4 reference manager, we were able to detect and eliminate those articles that were duplicated. Data extraction was performed independently by two reviewers (R.G.C. and M.A.C.), who resolved inconsistencies through discussion. We used a data extraction form to collect information on study objectives, study design, study population and main findings.

### Data analysis

For each study, we report the outcomes analysed as reported in the paper. We also report data sources, sample size, survey design (cross-sectional, longitudinal, or time series) and population studied (gender, age group, urban/rural, and socioeconomic status). We pay special attention to report the evaluation design, i.e., the analytical strategy used to address potential biases, identify causality, and the comparison group.

Most of the selected studies analysed several outcomes. Thus, we first identified six main categories of outcomes and ordered them following a continuum of care framework: healthcare utilization, screening, treatment, testing/monitoring, health outcomes (morbidity or mortality) and financial protection. We also identified subcategories of outcomes, such as specific diseases, age groups or financial protection indicators, within each of the six broad categories.

Because most of the papers analysed multiple outcomes, we graphically displayed the number of outcome subcategories and whether the results showed a positive or no impact. Finally, as studies could find positive impacts for some outcomes and not for others, we classified and displayed the number of papers that found “positive impact”, “no impact” or “mixed impact”. For data analysis, we used Excel^®^ version 2009 for Microsoft^®^.

## Results

**Fig. 1:**
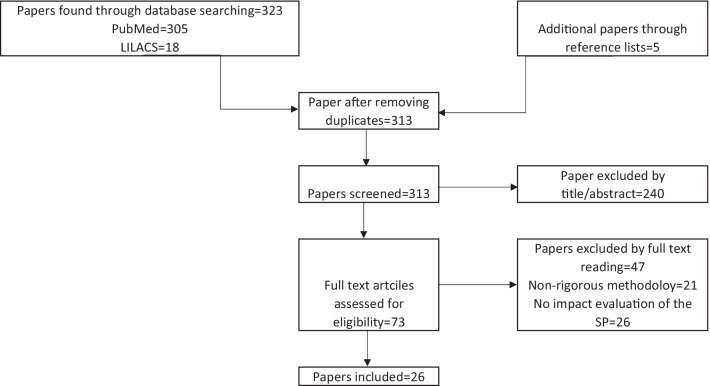
Analytical sample

The initial search identified 323 citations, from which we excluded 15 duplicates. We then reviewed titles and abstracts of the remaining 313 papers and excluded 240 studies that were not evaluations of the SP. We reviewed the full text of the remaining 73 articles to assess their impact evaluation methodology. After reading the full texts, we excluded 47 articles that failed to use methods to address potential self-selection biases and 26 that were not impact evaluations of the SP. We included 26 studies in the final selection, as shown in Fig. 1.

Table 1 summarizes the 26 papers included in the review by outcomes analysed, data sources, population studied, evaluation design and the comparison group. The most frequent data sources used were the National Health and Nutrition Surveys, cross-sectional surveys collected at different times (12 studies), the National Income and Expenditure Surveys, and cross-sectional surveys collected every 2 years (six studies). From the 26 selected papers, 22 relied on cross-sectional surveys, four on longitudinal data, and one used time series. To address the potential biases associated with the voluntary affiliation to the SP, the majority of the studies used propensity score matching (13 studies), instrumental variables (nine studies) or both methods combined (two studies). Concerning comparison groups, 17 used uninsured households, six used uninsured and social security, and three used social security beneficiaries.

**Table 1:** Description of the selected studies evaluating the impact of the Seguro Popular

Author	Outcome	Data	Population	Evaluation design	Group of comparison
Knox 2018 [15]	– General physical exams – Cervical cancer screening – Diabetes screening	– Urban Evaluation Survey (ENCERLUB, 2009 and 2014)^b^	– 23,599 individuals living in urban areas	Two-stage least squares (2SLS) with instrumental variables	– Uninsured
Rivera-Hernández 2019 [16]	– Pap smears – Mammography/clinical examination – Diabetes screening – Hypertension screening	– National Health and Nutrition Surveys (ENSANUT, 2000, 2006, 2012)^a^	– 17,640 adults aged 50 to 75 years	Two-stage least squares (2SLS) with instrumental variables, with fixed effects (pseudo panel from ENSANUT)	– Uninsured
Parker 2018 [17]	– Utilization and diagnostic tests – Receiving treatment: for hypertension, diet for diabetes, taking insulin for diabetes	– Longitudinal Mexican Health and Aging Study (2001–2012)^b^	– 15,186 adults, 50 years old or older	Difference-in-difference propensity score matching estimators	– Uninsured
Servan-Mori 2017 [18]	– Antenatal care cascade	– National Demographic Dynamics Survey (ENADID, 2009)^a^	– 14,414 women aged 15 to 50 years	Propensity score matching	– Uninsured
Servan-Mori 2015 [19]	– Access to prescribed medicines	– National Health and Nutrition Survey (ENSANUT 2012)^a^	– 6123 users of outpatient services	Two-stage least squares (2SLS) with instrumental variables	– Uninsured
Servan-Mori 2015 [20]	– Timely first antenatal visit (up to third month of gestation) and attendance at four antenatal visits	– National Health and Nutrition Survey, (ENSANUT, 2012)^a^	– 6175 women aged 14–49	Propensity score matching	– Uninsured
Sosa-Rubí 2009 [21]	– Access to laboratory tests, visits for diabetes control, treatment with any drug, number of control tests/month	– National Health and Nutrition Survey (ENSANUT, 2006)^a^	– 1491 adults with diabetes	Propensity score matching	– Uninsured
Sosa-Rubí 2009 [22]	– Access to obstetrical services	– National Health and Nutrition Survey (ENSANUT, 2006)^a^	– 3890 women who delivered babies during 2001–2006	Multinomial choice model with a discrete endogenous variable	– Non-SP-accredited clinic – Private
Bleich 2007 [23]	– Coverage of antihypertensive treatment – Coverage of antihypertensive treatment with control of blood pressure	– National Health and Nutrition Survey, (ENSANUT 2005)^a^ – Mexican National Registry of Health infrastructure	– 4032 adults with hypertension	Propensity score matching	– Uninsured
Arenas 2015 [24]	– Consultations and hospitalization	– Mexican Family Life Survey 2002 and 2015 (ENNViH, 2002 and 2015)^b^	– 6063 households	Propensity score matching	– Uninsured
Nikoloski 2018 [25]	– Out-of-pocket and catastrophic health spending	– National Health and Nutrition Survey (ENSANUT, 2006 and 2012)^a^	– 45,837 households in 2006 – 50,023 households in 2012	Two-stage least squares (2SLS) with instrumental variables	– Social security
García-Díaz 2018 [26]	– Out-of-pocket health spending	– National Income and Expenditure Survey (ENIGH, 2010)^a^	– 11,117 households	Propensity score matching	– Uninsured
Serván-Mori 2018 [27]	– Monetary and nonmonetary health service consumption	– National Income and Expenditure Survey (ENIGH, 2012)^a^	– 7040 households	Two-stage least squares (2SLS) with instrumental variables	– Social security – Uninsured
Knaul 2018 [28]	– Out-of-pocket and catastrophic expenditures	– National Income and Expenditure Surveys 2004–2012^a^	– 109,513 households	Propensity score matching	– Social security
Doubova 2015 [29]	– Access to healthcare – Catastrophic health-related expenditures	– National Health and Nutrition Survey (ENSANUT, 2012)^a^	– 18,847 older adults, 13,180 households that have an elderly member	Propensity score matching	– Social security – Uninsured
Ávila-Burgos 2013 [30]	– Out-of-pocket and catastrophic health spending	– National Health and Nutrition Survey (ENSANUT, 2012)^a^	– 12,250 households	Propensity score matching	– Uninsured
Wirtz 2012 [31]	– Out-of-pocket health spending	– National Income and Expenditure Survey (ENIGH, 2008)^a^	– 28,260 households	Propensity score matching and instrumental variables	– Uninsured – Social security – Mixed affiliations
Sosa-Rubí 2011 [32]	– Out-of-pocket and catastrophic health spending	– Seguro Popular evaluation Survey (2005–2008)^a^	– Rural cohort: 29,000 households – Urban cohort: 6000 households	Fixed effects with instrumental variables	– Uninsured
García-Díaz 2011 [33]	– Out-of-pocket health spending	– National Income and Expenditure Survey (ENIGH, 2006)^a^	– 3665 SP affiliates – 7638 “Oportunidades” affiliates, – 1506 SP and “Oportunidades” affiliates – 43,539 without any affiliation	Propensity score matching and instrumental variables	– Oportunidades – Uninsured
Galárraga 2010 [34]	– Out-of-pocket and catastrophic health spending	– National Health and Nutrition Survey (ENSANUT, 2006)^a^ – SP Impact Evaluation Survey (2005–2006)^a^	– SP Impact Evaluation Survey: 4033 SP-insured households and 16,759 uninsured households – ENSANUT: 4440 SP-insured households and 16,376 uninsured households	Two-stage least squares (2SLS) with instrumental variables	– Uninsured
King 2009 [35]	– Out-of-pocket and catastrophic health spending	Survey designed by the authors^b^	– 16,256 households – 1205 households enrolled – 15,051 households unenrolled	A matched-pair cluster-randomized experiment	– Uninsured
Hernández-Torres 2008 [36]	– Catastrophic health spending	– Seguro Popular evaluation Survey, 2002^a^	– 2158 households – 482 were affiliated with the SP and 1676 had no affiliation	Two-stage least squares (2SLS) with instrumental variables	– Uninsured
Rivera-Hernandez 2016 [37]	– Diabetes treatment and care process indicators – Hypertension treatment and care process indicators	– National Health and Nutrition Survey (ENSANUT, 2000, 2006 and 2012)^a^	– 3015 older adults aged over 50 diagnosed with diabetes – 5307 older adults aged over 50 diagnosed with hypertension	Two-stage least squares (2SLS) with instrumental variables, with fixed effects	– Uninsured
Celhay 2019 [38]	– Out-of-pocket expenses – Health outcomes in children	– Data sets from the National Institute of Statistic and Geography^c^	– 11.39 million children born and living in Mexico	Difference-in-difference using interrupted time series and fixed effects	– Social security – Uninsured
Grogger 2012 [39]	– Out-of-pocket expenses	– National Income and Expenditure Survey (ENIGH, 2008)^a^	– 31,040 households in rural areas – 56,696 households in urban areas	Propensity score matching and instrumental variables	– Uninsured
Gutierrez 2018 [40]	– Out-of-pocket expenses	– National Health and Nutrition Survey (ENSANUT, 2012)^a^	– 44,000 households with at least one member with diabetes, hypertension, or both	Propensity score matching	– Uninsured – Social security

Data source design: ^a^cross-sectional, ^b^longitudinal, ^c^time series

Additional file 1 shows the heat map with results from the SURE quality instrument. From the 11 items, the average score for the 26 papers selected is 10.2, ranging from 8 to 11. All studies comply with the following aspects: study question; settings, locations and dates; participant selection, characteristics of participants; appropriate outcome and exposure measures; sample size; adequate description of the methods and results. Twelve papers had lower scores in the description of the study design in the abstract, and nine papers failed to report sponsorship/conflicts of interest.

From the selected studies, nine evaluated healthcare utilization such as infant, perinatal and general healthcare visits. Three focused on screening for hypertension, diabetes and gynaecological or prostate cancer. Five assessed access to hypertension, diabetes or general care, and three papers focused on diabetes diagnosis. One study evaluated newborn and infant mortality and child development. Finally, 16 studies analysed the impact of the SP on financial protection, including out-of-pocket expenditures, impoverishing spending or catastrophic health expenses.

Table 2 shows the number of outcomes for each of the main categories and subcategories. The 26 studies analysed 137 outcomes**:** 65 on financial protection (47%), 30 on healthcare utilization (22%), 19 on treatment (14%), 14 on screening (10%), five for testing or monitoring (4%) and only four on morbidity or mortality (3%). From all outcomes, 66 were statistically significant, and 71 reported no significant impact.

**Table 2:** Impact of the Seguro Popular by broad category of outcomes and specific outcomes

Outcomes broad categories		Outcomes subcategories		Statistically significant effect	Not statistically significant effect
Utilization	30	General healthcare utilization	17	6	11
		Infant healthcare utilization	3	0	3
		Perinatal care	10	6	4
Screening	14	Hypertension screening	3	2	1
		Diabetes screening	4	2	2
		Gynaecological screening	5	2	3
		Prostate cancer screening	2	1	1
Treatment	19	Hypertension treatment	8	0	8
		Diabetes treatment	10	3	7
		General healthcare treatment	1	1	0
Testing/monitoring	5	Diabetes follow-up test	5	4	1
Morbidity/mortality	4	Newborn mortality	2	1	1
		Infant mortality	1	1	0
		Child development	1	1	0
Financial protection	65	Out-of-pocket expenses	46	27	19
		Impoverishing spending	1	1	0
		Catastrophic health expenses	18	8	10
Total	137		137	66	71

**Fig. 2:**
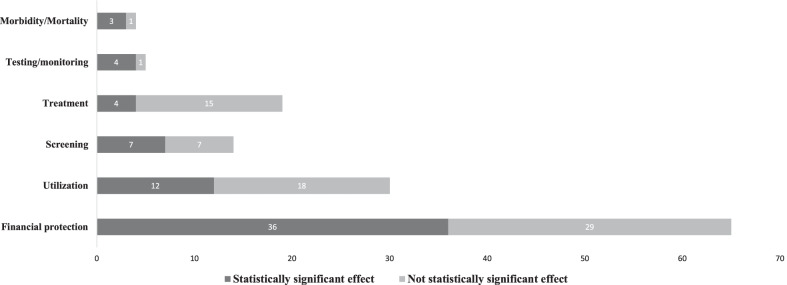
Impact of the Seguro Popular by outcome categories

Figure 2 shows the number of outcomes reported in the studies by six broad categories. The figure shows the number of statistically significant and nonsignificant results for each category. From the 65 outcomes on financial protection, 55% were statistically significant. Concerning healthcare utilization, 40% of the 30 outcomes analysed were significant. For screening, from the 14 outcomes analyzed, 50% were significant. The authors found statistically significant effects in 21% of the 19 treatment outcomes, four of the five monitoring and testing outcomes, and three of four morbidity and mortality outcomes.

Finally, Additional file 2 shows the number of studies by type of result. From the 26 studies, eight found no significant effect in any of the outcomes analysed by those studies, six reported statistically significant effects in all outcomes, and 12 found mixed results (statistically significant effects for some outcomes but nonsignificant results for others). For more details on the specific results by outcome/study see Additional file 3.

## Discussion

We conducted a systematic review of published studies that evaluated the impact of the SP in Mexico between 2007 and 2019. We found 26 papers that used rigorous methods for impact evaluation and 47 that evaluated the SP but were excluded because the methods used failed to address the potential selection bias, as affiliation to SP was voluntary. From the 26 papers included, more than half of the studies estimated the impact of the SP on financial protection. One paper assessed the effect of the SP on morbidity and mortality for neonates, and the rest of the papers estimated the impact on healthcare utilization, screening, treatment and testing/monitoring. The 26 studies analysed 137 outcomes**:** 65 on financial protection (47%), 30 on healthcare utilization (22%), 19 on treatment (14%), 14 on screening (10%), five for testing or monitoring (4%), and only four on morbidity or mortality (3%).

We found a wide variation in the populations studied by age, sex and urbanicity. About 85% of the papers relied on cross-sectional surveys. Most studies used either propensity score matching or instrumental variables to address self-selection bias, as affiliation to SP is voluntary. Sixty-five percent of the studies compared individuals enrolled in the SP with unenrolled groups, and 23% compared with unenrolled and social security affiliates. The rest used the population insured by social security institutions.

The papers analysed a great variety of outcomes, and their results showed substantial heterogeneity in the impact. Overall, 55% of the outcomes analysed showed a positive impact, and 45% no impact. We found evidence of a positive impact on three morbidity/mortality outcomes, 14 healthcare utilization outcomes, and 18 for financial protection. Concerning screening and access to treatment, the studies documented positive results on eight outcomes. Our results show that 50% of the papers reported mixed results, 31% positive impacts, and 19% no effects in all outcomes analysed.

We found more significant impacts on financial protection than on treatment, screening or utilization. This result is consistent with a key feature of the SP as a funding mechanism to increase access to a predetermined set of services. However, much weaker effects on healthcare utilization and health outcomes have been explained previously by the SP operation as a decentralized programme by state governments, with heterogeneity in the capacity to provide care [9]. We cannot rule out the possibility of flawed study designs as well; however, our results on the papers' quality suggest this is not the most likely cause. For the uninsured population that used private services before the SP, evidence on the use of private services suggests that it reduced their financial burden [10]. Moreover, there was no significant expansion of the supply of services following the establishment of the SP [10, 11]. Therefore, it is possible that the SP did not expand the access to services to more people, but rather it became an affordable alternative for people who already had access to healthcare through private services.

To our knowledge, this is the first systematic review of rigorous impact evaluations of the Mexican SP. Other systematic reviews of similar financial protection mechanisms in other countries [12] have reported impacts on financial protection, health services utilization and health outcomes. Some studies found positive results with reduced out-of-pocket expenses, but negative results for catastrophic expenses [13]. In contrast, an Indian study found no protective effect on out-of-pocket spending but observed reduced mortality in insured compared to uninsured households [14]. A study that evaluated the impact of similar programmes in Asia and Africa found increased use of medical services and financial protection by reducing out-of-pocket expenses, but little evidence of a positive effect on the quality of care, and inconclusive effects regarding the empowerment of communities [12]. However, in contrast to our study, many of these reviews included qualitative or mixed-methods studies, and none considered rigorous impact evaluation methods as an inclusion criterion. Nonetheless, the reviews also found mixed results such as reductions in out-of-pocket expenses but adverse effects on catastrophic expenses [13]. Others showed no impact on financial protection but found reductions in mortality [14].

By 2018, affiliation to the SP reached 53.5 million people, 44.7% of the total population [5]. Affiliation, however, was not followed by an equivalent increase in supply [11]. A recent paper on this topic showed that despite this massive increase in coverage, 46.4% of affiliates to the SP reported using private services in 2018. These private options include medical offices in pharmacies that are not regulated; the quality of services has not been documented and is associated with higher out-of-pocket expenses [10].

One limitation of the study is that we could not summarize the findings using meta-analyses because of the considerable heterogeneity in outcomes and how they were measured (see Additional file 3).

## Conclusions

In conclusion, the published papers that estimated the effects of the SP show considerable impact heterogeneity. Despite including only rigorous evaluations in our review, researchers were able to find statistically significant effects in 55% of the financial protection outcomes and 40% for healthcare utilization outcomes evaluated. Furthermore, except for one paper, no evidence exists on the impact of the SP on health outcomes or mortality.

## Supplementary Information

**Additional file 1.** Specialist Unit for Review of Evidence (SURE) that evaluates the quality of 11 key features for each of the 26 selected papers that evaluated the Seguro Popular.

**Additional file 2.** Heterogeneity in the impact of the Seguro Popular: number of studies with statistically significant effects, not statistically significant effects or mixed effects from the 26 selected papers that evaluated the Seguro Popular.

**Additional file 3.** Specific results by outcome/paper from the 26 papers that evaluated the Seguro Popular.

## Data Availability

The datasets used and/or analysed during the current study are available from the corresponding author on reasonable request.

## Abbreviations

SP: Seguro Popular (Popular/Public Health Insurance)
UHC: Universal health coverage
LILACS: Literatura Latinoamericana de Información en Ciencias de la Salud (Latin American and Caribbean Literature in Health Sciences)
SURE: Specialist Unit for Review Evidence
