# Supplementary figures and images for: A systematic review of the literature on the impact of the Seguro Popular
Source: Health Res Policy Syst. 2022 Apr 18;20:42. doi: 10.1186/s12961-022-00839-w (PMC9014564; doi:10.1186/s12961-022-00839-w)

## Additional file 2: Heterogeneity in the impact of the Seguro Popular

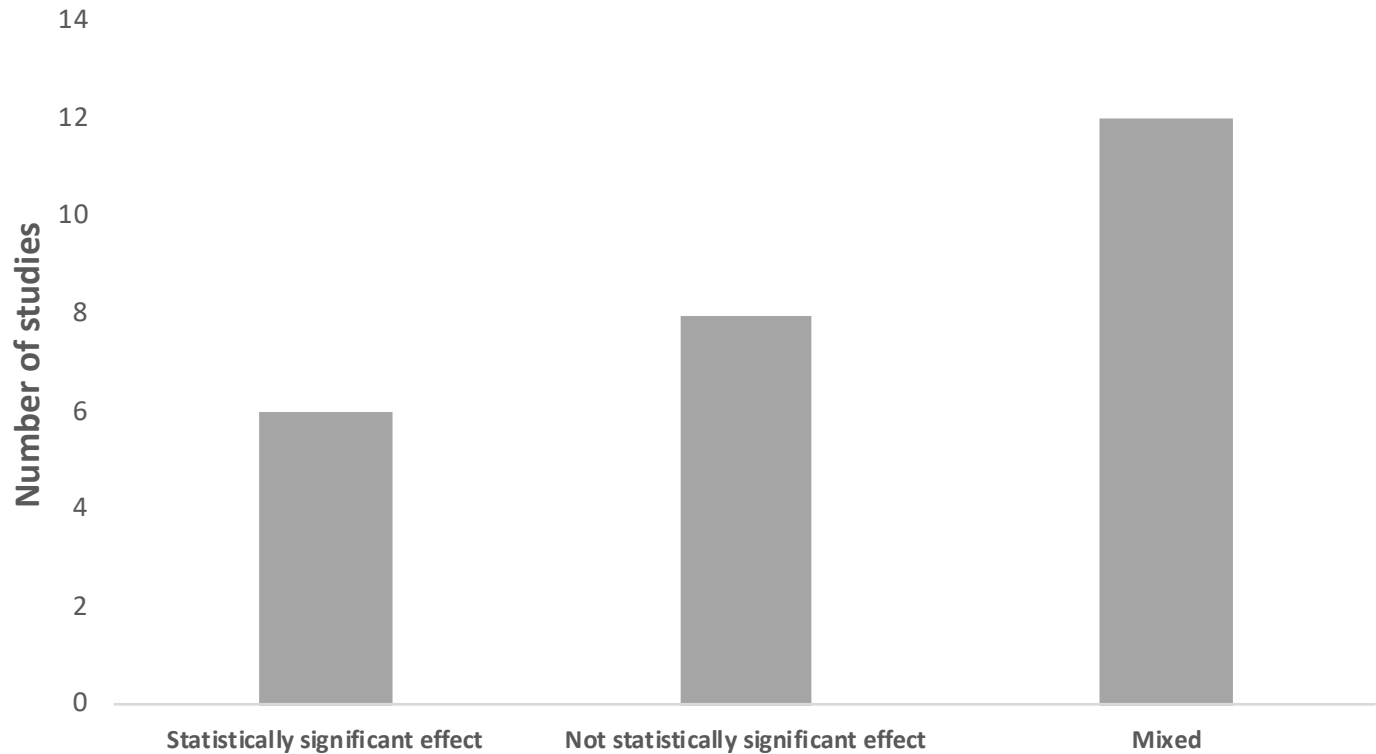

Supplement: Supplementary file 2 — Additional file 2. Heterogeneity in the impact of the Seguro Popular: number of studies with statistically significant effects, not statistically significant effects or mixed effects from the 26 selected papers that evaluated the Seguro Popular. [file 12961_2022_839_MOESM2_ESM.pdf]
